# Getting our house in order: an audit of the registration and publication of clinical trials supported by the National Institute for Health Research Oxford Biomedical Research Centre and the Musculoskeletal Biomedical Research Unit

**DOI:** 10.1136/bmjopen-2015-009285

**Published:** 2016-03-02

**Authors:** A C Tompson, S Petit-Zeman, B Goldacre, C J Heneghan

**Affiliations:** 1 Nuffield Department of Primary Care Health Sciences, The Centre for Evidence-Based Medicine, University of Oxford, Oxford, UK; 2 National Institute of Health Research Oxford Biomedical Research Centre and Unit, Oxford University Hospitals NHS Trust, Oxford, UK

**Keywords:** PUBLIC HEALTH, STATISTICS & RESEARCH METHODS

## Abstract

**Objectives:**

To audit the proportion of clinical trials that had been publically registered and, of the completed trials, the proportion published.

**Setting:**

2 major research institutions supported by the National Institute of Health Research (NIHR).

**Primary and secondary outcome measures:**

The proportion of trials reporting results within 12 months, 24 months and ‘ever’. Factors associated with non-publication were analysed using logistic regression.

**Inclusion criteria:**

Phases 2–4 clinical trials identified from internal documents and publication lists.

**Results:**

In total, 286 trials were identified. We could not find registration for 4 (1.4%) of these, all of which were completed and published. Of the trials with a registered completion date pre-January 2015, just over half (56%) were published, and half of these were published within 12 months (36/147, 25%). For some trials, information on the public registers was found to be out-of-date and/or inaccurate. No clinical trial characteristics were found to be significantly associated with non-publication. We have produced resources to facilitate similar audits elsewhere.

**Conclusions:**

It was feasible to conduct an internal audit of registration and publication in 2 major research institutions. Performance was similar to, or better than, comparable cohorts of trials sampled from registries. The major resource input required was manually seeking information: if all registry entries were maintained, then almost the entire process of audit could be automated—and routinely updated—for all research centres and funders.

**Strengths and limitations of this study**

- To our knowledge, this is the first published audit of clinical trial registration and publication performance for two major research institutions.
- By using internal records, it was possible to assess if there were any unregistered, unpublished trials: many other studies of trial registration have worked from published papers, making it impossible to assess this.
- There was a limited response from investigators when contacted to supplement the limited information contained within the internal records.
- We have produced and shared resources to facilitate similar audits elsewhere.

## Introduction

The public registration of clinical trials, and the timely publication of their results, are widely accepted as scientific and moral obligations. Guidelines issued by a range of regulatory bodies, including the WHO,^1^ the World Medical Association,^2^ the US Food and Drug Administration (FDA)^3^ and the European Commission^4^ have encouraged and latterly mandated these activities for funders and triallists. Since 2013, the UK's Health Research Authority will explicitly not approve applications to conduct clinical trials that are not registered.^5^

The AllTrials Campaign developed in response to professional, public and patient concern regarding the lack of progress in this area, and calls for all trials to be registered and reported.^6^ It is estimated that about a half of all trials go unreported^7^ and two cohort studies from 2012 to 2015 have found that only a fifth of trials registered on clinicaltrials.gov reported results within 1 year of completion.^8^ ^9^ This is despite the reporting of results within 12 months of completion being mandatory for all trials that fall under the FDA Amendments Act legislation.^3^ Estimates of non-registration are harder to collect, since unregistered and unpublished trials are hard to identify, but one cohort study found that only half of all trials published in major medical journals were properly registered, and a quarter were not registered at all.^10^

In 2015, the WHO published a landmark position statement, requiring all trials to make their methods and results available.^1^ Specifically the WHO stated that results from clinical trials should be publicly reported within 12 months of their end, and that results from previously unpublished trials should be made publicly available. They also stated that organisations and governments should implement measures to achieve this.

We have previously suggested that research organisations and companies should routinely audit themselves to ensure all trials’ methods and results are made publicly accessible.^11^ Inspired by the AllTrials Campaign, the Patient Involvement Working Group at the National Institute of Health Research (NIHR) Oxford Biomedical Research Centre (BRC) and the NIHR Oxford Musculoskeletal Biomedical Research Unit (BRU) suggested that the BRC/BRU's own track record of registering clinical trials and publishing their results should be scrutinised, and funded this work.

## Methods

We aimed to assess the proportion of clinical trials that had been publically registered and the proportion of completed trials that had been published. We also aimed to assess the proportion published within 12 months of trial completion.

We undertook a retrospective audit of all clinical trials receiving support from the NIHR Oxford BRC and/or BRU. These organisations are collaborations between the University of Oxford and the Oxford University Hospitals National Health Service (NHS) Trust. As recognised centres of research excellence, they have been awarded over £160 million pounds of public funds via the NIHR since their inception in 2007.^12^ ^13^ The BRC and BRU fund-specific projects (in whole or in part) and also invest in research staff and infrastructure to improve the translation of basic scientific developments into clinical benefit. Therefore, the trials included in this audit may have been funded wholly or in part by the BRC/BRU and/or have received input from BRC/BRU-funded researchers and/or been conducted in BRC/BRU-supported facilities.

We focused the audit on phases 2, 3 and 4 clinical trials as earlier phase trials are exempt from the requirements to be registered and published.^3^ Individual trials that covered both phases 1 and 2 were included. We conducted the registration and publication searches in January 2015 and used the BRC/BRU annual activity reports submitted to the NIHR between 2009 and 2014 to compile a list of trials. We cross-checked this list using a publication list (BRC) and publically available annual reports (BRU). We undertook the audit using four distinct steps:

### Step 1: creating the inventory of clinical trials

**Figure 1:**
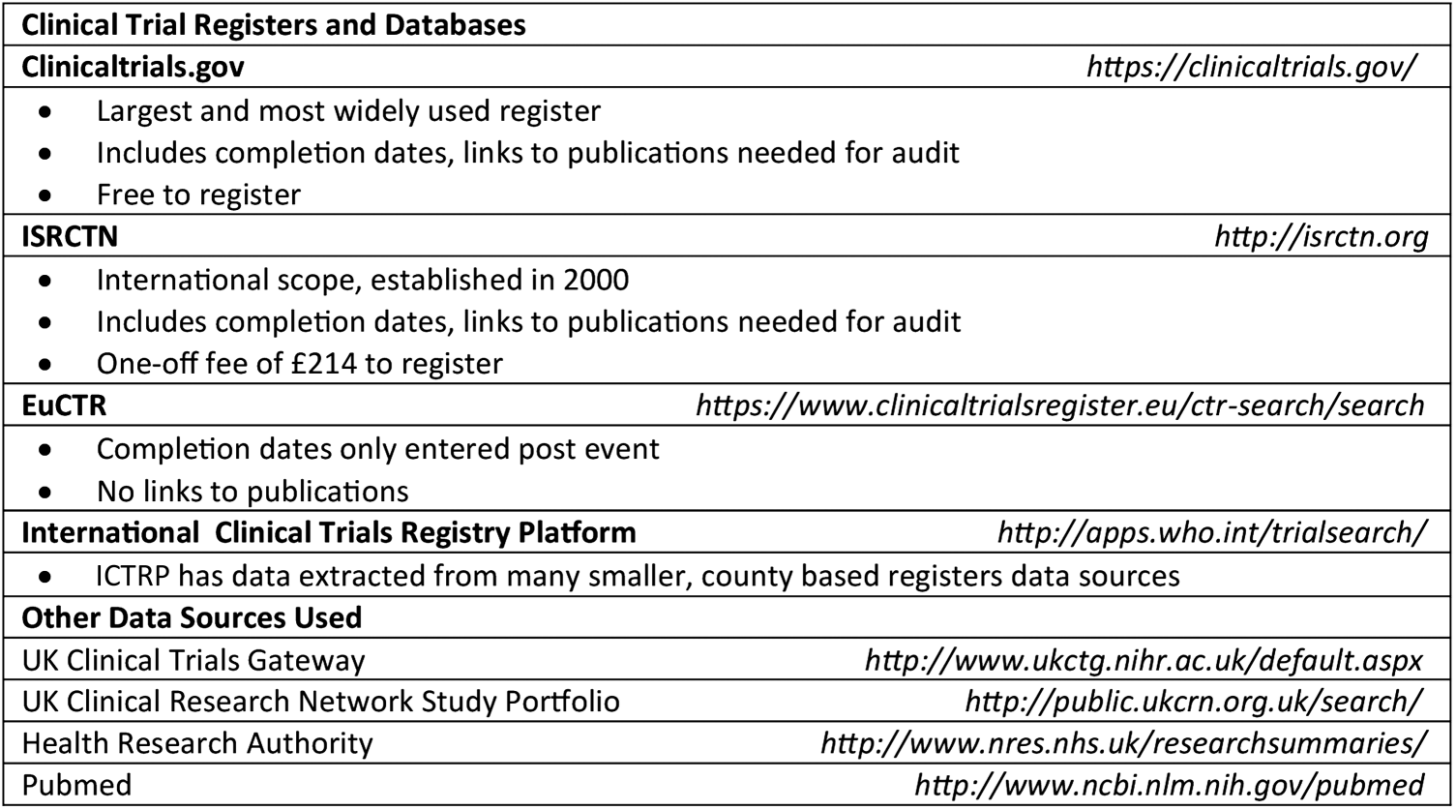
Hierarchy of sources used to verify records. EuCTR, EU Clinical Trials Register; ICTRP, International Clinical Trials Registry Platform; ISRCTN, International Standard Randomised Controlled Trial Number.

One researcher (ACT) reviewed the details of the projects contained within the annual activity reports to remove duplicates and then assessed eligibility by determining study type and clinical trial phase, where applicable. Records were supplemented with data in publically accessible registries/databases if needed (figure 1). Eligibility queries were resolved by discussion between CJH and ACT: if this was not possible, we sent an email enquiry to the project lead investigator.

### Step 2: populating the inventory and assessing registration

One researcher (ACT) searched the four major clinical trial registries using the full trial title or, if not available, combinations of other identifying features (eg, drug name and target condition; figure 1). The registries were searched in a specific order for pragmatic reasons based on their size and their inclusion of the information required for the audit. We contacted the trial investigator if we could not locate a registry entry for their project. We populated the audit inventory with the following variables, extracted directly from the register where possible: official title, abbreviated title, trial identification numbers, sponsor details, funding bodies, trial status, completion date (anticipated or actual), presence of any uploaded results and any listed publications.

### Step 3: verification of the inventory

One researcher (ACT) checked the completeness of the inventory using a list of BRC-supported research publications and publically available BRU annual reports. We asked BRU investigators to supplement the details of the trials mentioned due to the limited information available in the annual reports. A list of phases 2, 3 and 4 trials was produced and cross-referenced against the inventory.

### Step 4: assessing publication

Based on our inventory, one researcher (ACT) searched for publications for clinical trials that gave a completion date before January 2015. For trials registered on clinicaltrials.gov, the overall study completion date was used; if not entered, the primary outcome completion date was used instead. In addition to publications listed on the registry and results posted in tabular format to clinicaltrials.gov, we also searched PubMed (http://www.ncbi.nlm.nih.gov/pubmed) for articles arising from the completed trials. Those describing the protocol design or analysis plan were excluded. Finally, if it was not possible to locate a publication for a clinical trial with a completion date of before January 2014, we contacted the BRC/BRU investigators directly. This cut-off was chosen because the European Union (EU; since 2014) and the FDA (since 2007) require clinical trials to post results within 12 months following the end date of the trial.^3^ ^4^

### Data analysis

Analysis was conducted with Stata MP V.14. The data were described, and logistic regression used to examine trial characteristics associated with slow/non-publication. A logistic regression model was built using ‘sponsor type’, ‘phase’ and ‘sample size’ as explanatory variables. This model was constructed prior to analysing the data, rather than by using forward selection, and used widely available variables from registry entries, so that future audits can use the same methods and code to produce comparable results.

## Results

Based on step 1 of our method, we assessed 1255 projects for eligibility and compiled an initial inventory of 247 clinical trials: table 1 shows that 215 were BRC supported, 29 were BRU supported and 3 trials received support from both organisations.

**Table 1:** The number and registration status of Biomedical Research Centre (BRC)/Biomedical Research Unit (BRU)-supported clinical trials included in the audit

	BRC (n=215)		BRU (n=29)		Both (n=3)		Total (n=247)	
Inventory—step 1	n	Per cent	n	Per cent	N	Per cent	n	Per cent
Unregistered	2	0.9	0	0	0	0	2	0.8
Verification—step 3	BRC (n=34)		BRU (n=5)		Both (n=0)		Total (n=39)	
	n	Per cent	n	Per cent	N	Per cent	n	Per cent
Unregistered	2	5.9	0	0	–	–	2	5.1
Combined	BRC (n=249)		BRU (n=34)		Both (n=3)		Total (n=286)	
	n	Per cent	n	Per cent	N	Per cent	n	Per cent
Unregistered	4	1.6	0	0	0	0	4	1.4

The verification method, outlined in step 3, identified a further 39 (16%) trials. Therefore, the total number of trials supported by the BRC and/or BRU and included in the audit was 286 with over 217 000 participants worldwide. Of these, 90 (31%) were sponsored by Oxford (University and/or Oxford University Hospitals NHS Trust) and 108 (38%) were sponsored by industry. The remainder were sponsored by other academic, charitable or non-industry sources. (The sponsor is responsible for the initiation, management and/or financing of a clinical trial.)

### Registration

We could not find public registry entries for six trials identified (4 in step 1 and 2 trials in step 3), and so we contacted these investigators. One was in fact a duplicate of an already identified trial and another was registered on the EU Clinical Trials Register (EuCTR) database, although no results had been produced when we searched the register.

Therefore, in total, we identified 4/286 (1.4%) unregistered trials, all of which had been completed and published: One was an e-health intervention described as a pilot study; another was a surgical trial conducted in 2009 at which time the lead investigator (incorrectly) believed it did not require registration. The final two were oncology trials which, although started in the early 2000s, were published in 2011 and 2014, after launch of the International Committee of Medical Journal Editor's policy requiring clinical trial registration.^14^

### Publication

One hundred and forty-seven (147) trials had a pre-January 2015 completion date (118 identified via step 1 and 29 identified via step 3).

We found academic publications reporting results for 65 (44%); 3 (2.0%) had summary results uploaded on the clinical trials register; 12 trials (8.2%) had both.

**Figure 2:**
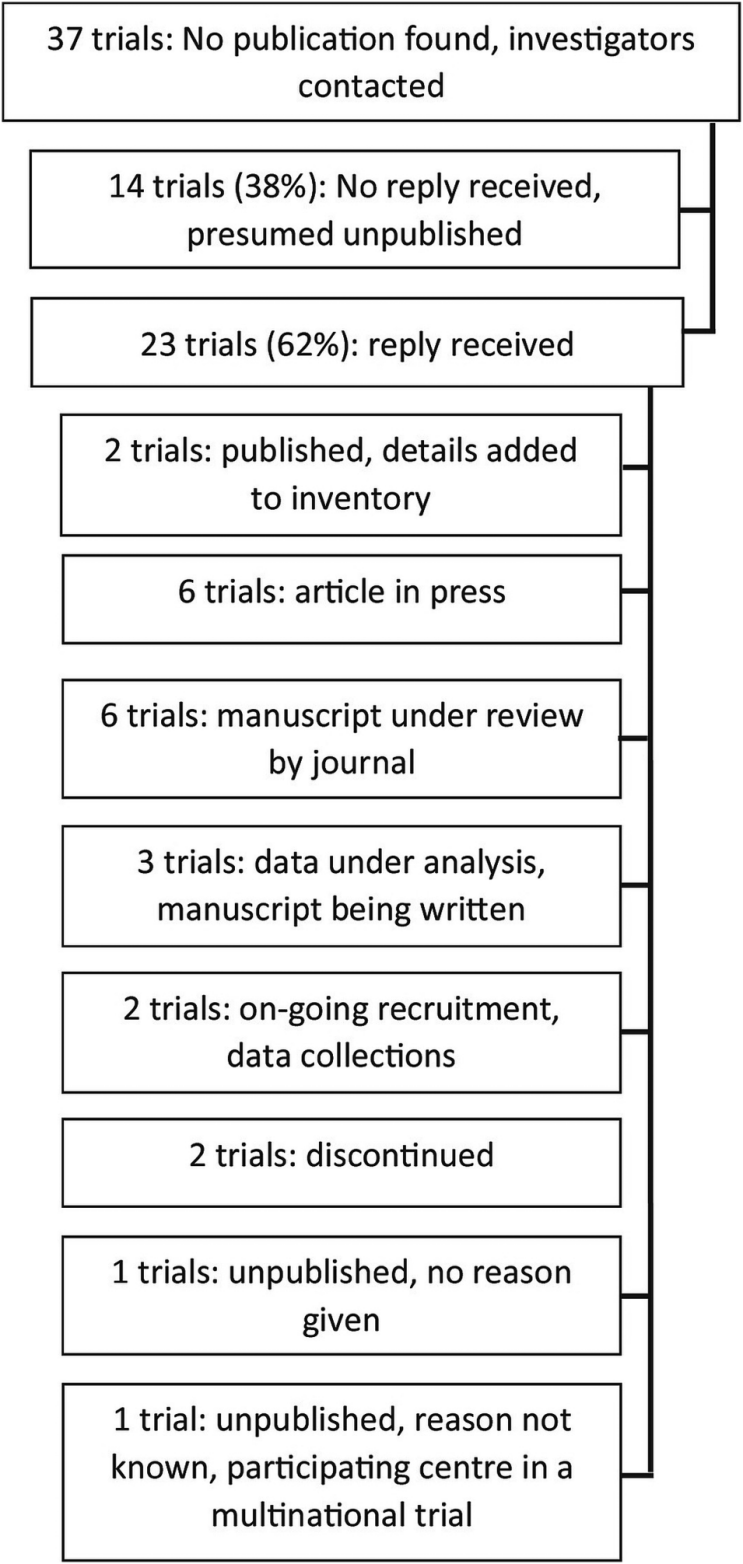
Audit flow chart of clinical trials that were overdue publication.

We contacted investigators for the 37 (33%) of the 112 trials with a completion date of pre-January 2014, 12 months prior to the audit date, for which no publication had been identified and where publication was therefore overdue (figure 2). No reply was received from 14 triallists, and these projects were therefore classified as unpublished. Of the 23 trials for which responses were received: 17 (74%) trials were unpublished due to delays; 6 were in press; 6 had manuscripts under journal review; 3 trials were undergoing analysis; 2 reported they were still collecting data; 2 trials had been discontinued; and for 2 trials the reason for non-publication was not known by the Oxford-based collaborator or not given. The remaining two investigators supplied publication details that our search had missed and these were added to the audit database.

Overall, of the trials with a pre-January 2015 completion date, 82 of the 147 (56%) were published or had uploaded summary results, with the BRC and BRU achieving 54% and 67% publication, respectively (table 2).

**Table 2:** Publication status of Biomedical Research Centre (BRC)/Biomedical Research Unit (BRU)-supported trials with a registered pre-January 2015 completion date

	BRC (n=101)		BRU (n=17)		Total (n=118)	
Inventory—step 1	n	Per cent	n	Per cent	n	Per cent
Published (within 12 months of trial completion)	17	16.8	4	23.5	21	17.8
Published (over 12 months of trial completion)	29	28.7	7	41.2	36	30.5
Unpublished	55	54.4	6	35.3	61	51.7
Verification—step 3	BRC (n=28)		BRU (n=1)		Total (n=29)	
	n	Per cent	n	Per cent	n	Per cent
Published (within 12 months of trial completion)	14	50.0	1	100	15	51.7
Published (over 12 months of trial completion)	10	35.7	0	0	10	34.5
Unpublished	4	14.3	0	0	4	13.8
Combined	BRC (n=129)		BRU (n=18)		Total (n=147)	
	n	Per cent	n	Per cent	n	Per cent
Published	70	54.2	12	66.7	82	55.7
Unpublished	59	45.7	6	33.3	65	44.2

**Figure 3:**
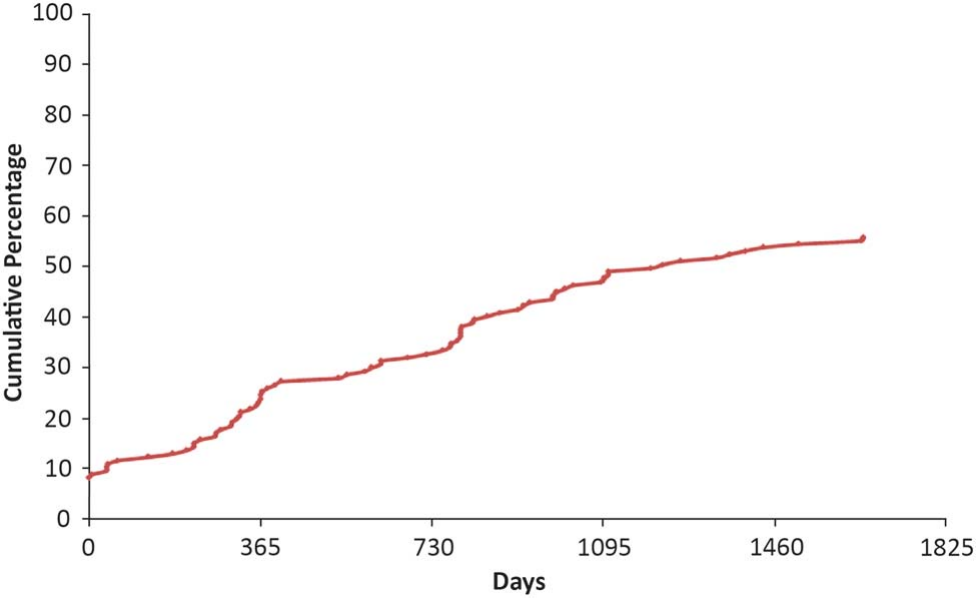
The time from registered completion date to publication of Biomedical Research Centre (BRC)/Biomedical Research Unit (BRU)-supported phases 2–4 trials.

**Figure 4:**
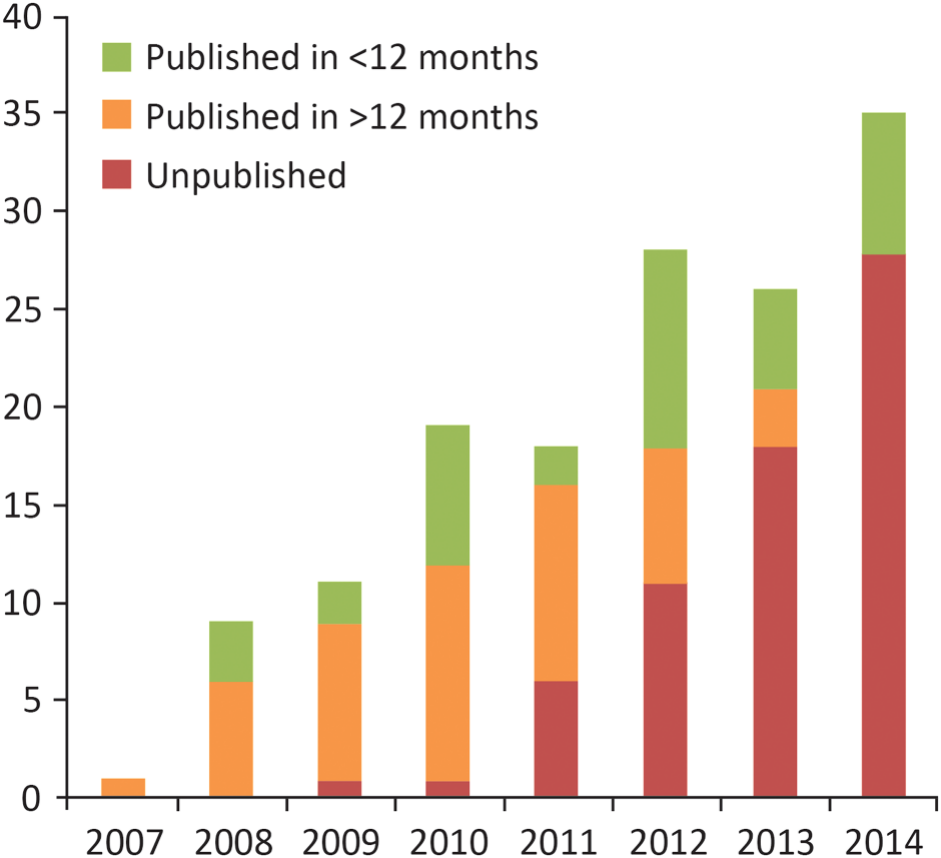
The publication status of Biomedical Research Centre (BRC)/Biomedical Research Unit (BRU)-supported phases 2–4 trials by registered completion year.

Publication or uploading of trial results within 12 months of the registered completion date was less common, as reported in figure 3: 36 of the 147 trials (25%) were published within 12 months and 48 (33%) within 24 months. The longest duration to publication was 54 months. Figure 4 shows that over the 7 years follow-up reported here, there has been no clear trend in the number of trials reporting results within 12 months of completion.

### Accuracy and completeness of registry data

Fourteen of the 282 registered trials were registered on EuCTR which does not require an expected completion date to be uploaded at the time of registration. A further 77 trials were registered on the ISRCTN register (known originally as the International Standard Randomised Controlled Trial Number but now broader in scope). This allows only one completion date to be registered, which is not ideal for trials with short-term and long-term follow-up.

Using the overall study completion date for our calculations, 12 trials appeared to have been published *earlier* than the completion date on their registry entry, giving a negative figure for publication delay. One publication described interim trial results while another five reported results for primary outcomes. Investigations into the reasons for early publication of the remaining trials found that registry data were either incorrect or out of date: one trial was discontinued, and the data collected were published early, but the trial was marked as ‘completed’ rather than ‘terminated’ and the completion date left unchanged; one trial was listed as a two-arm design, but on closer inspection the data were apparently two similar trials, at two different sites, with apparently incorrect use of trial identification numbers in the relevant documents; one trial registry entry erroneously contained a link to the results for a different trial of a similar design; two trials were registered as having follow-up time of 5–6 and 5 years, respectively, then published outcome data with median follow-up 4.3 and 3.8 years, respectively, but the registered completion dates remained unchanged; and one trial was entered onto two registries, with a completion date on ISRCTN 2 years earlier than that on clinicaltrials.gov.

Given these discrepancies, a random sample of 10 trials was selected from the list of trials with associated publications. Their outcomes, completion dates, sponsor and trial sites were compared between registry entry and publication, to assess whether any further trial publications on registry entries had been attached to the wrong trial on the registry: none in the sample had been.

### Factors associated with publication/non-publication of results

Table 3 presents the results of the logistic regression: no variables (sponsor type, trial phase or sample size) were found to be significantly associated with non-publication.

**Table 3:** Characteristics of published and unpublished trials

				OR			
		n	Published, %	Crude (95% CI)	p Value	Adjusted (95% CI)	p Value
Sponsor type	Non-industry	103	52, 49.51	1		1	
	Industry	44	27, 61.36	1.56 (0.76 to 3.20)	0.227	2.03 (0.88 to 4.69)	0.099
Phase	1, 2	12	5, 41.67	0.74 (0.21 to 2.67)	0.650	0.89 (0.24 to 3.33)	0.860
	2	49	24, 48.98	1		1	–
	2, 3	6	5, 83.33	5.21 (0.57 to 47.90)	0.145	5.13 (0.52 to 50.33)	0.160
	3	26	12, 46.15	0.89 (0.34 to 2.32)	0.816	0.68 (0.23 to 2.05)	0.492
	4	14	9, 64.29	1.87 (0.55 to 6.40)	0.316	2.10 (0.59 to 7.50)	0.256
	Not applicable	40	24, 60.00	1.56 (0.67 to 3.64)	0.301	1.95 (0.77 to 4.99)	0.161
Sample size (quartiles)	First (4–52)	34	17, 50.00	0.95 (0.38 to 2.38)	0.910	1.07 (0.41 to 2.77)	0.894
	Second (52–116)	39	20, 51.28	1	–	1	–
	Third (116–387)	36	19, 52.78	1.06 (0.43 to 2.63)	0.900	0.98 (0.36 to 2.64)	0.969
	Fourth (387–15 460)	37	23, 62.16	1.56 (0.63 to 3.89	0.340	1.48 (0.51 to 4.31)	0.474

## Discussion

### Summary of findings

It was feasible to conduct an internal audit of registration and publication of clinical trials in two major research institutions, covering almost 300 trials. Working from internal documents, we did not find any evidence of active clinical trials that were unregistered; however, we did identify four completed trials that had been published but not registered. The majority of completed trials were eventually published, but not all within 12 or even 24 months of completion. Lastly, some registry data were found to be incorrect or out of date.

### Strengths

To our knowledge, this is the first published audit of publication performance for major research institutions. By using internal records as a data source, it was possible to get good data on trial registration as well as publication: many other studies of completeness of registration have worked backwards from published papers, therefore making it impossible to identify and assess registration status of any unpublished trials.

Our audit builds on the foundations laid in Oxford 25 years ago by Easterbrook *et al*^15^ who tracked studies following their approval by the local research ethics committee and found evidence of publication bias. Since then, the requirements for clinical trial transparency and the supporting infrastucture have developed greatly, both prompting the need for—and enabling—audits such as ours.

### Limitations

The response rate from investigators when contacted with queries was poor, and consequently the eligibility of six projects listed in the BRC and BRU internal records remains unresolved. We hope that future response rates will improve as institutional audits of the registration and publication of clinical trials become the norm with trial registration and publication become indisputable steps in research for patient benefit. In addition, the audit did not examine the contents of trial publications, specifically whether the published outcomes match those prespecified in the registry entry, a known source of research bias.^10^ Similarly, the quality of the information entered by investigators into clinical trial registries was not scrutinised for all trials. Classifying non-pharmaceutical trials was difficult. As has been noted elsewhere, clearer guidance is needed on how to categorise trials (including pilot studies) and the requirements for their registration.^16^

### How do publication performance of trials supported by the NIHR Oxford BRC and BRU compare with other institutions?

A 2014 systematic review evaluated the publication rates of 22 cohorts of trials that had been identified via trial registries.^17^ These cohort studies were published between 2008 and 2013, and together included a range of medical specialties and over 16 000 trials. The proportion of trials published as peer-reviewed journal articles ranged from 13% to 90% with the review authors calculating that the weighted pooled proportion would be 54% after a minimum follow-up time of 24 months after trial completion (95% CI 42% to 66%, I^2^ 98.9%). There were 86 BRC/BRU trials with a pre-January 2013 completion date, thus allowing 24 months of follow-up time; of these, 67 (78%) had been published, exceeding the average performance in other published cohorts.

For timely publication, two cohort studies have been published looking at trials registered on clinicaltrials.gov to establish compliance with the FDA Amendment Act 2007, which requires publication for certain trials within 12 months of completion.^8^ ^9^ These two previous studies estimate publication rates at 12 months to be 22% and 13%, respectively, compared with 25% for our cohort. However, both previous studies exempted trials that were not covered by the FDA Amendment Act (such as trials on drugs that have not yet been licensed), and so may overestimate performance for timely publication across all trials when compared with the methods of our comprehensive audit.

Given the provenance of this study (the patient co-led and focused Patient Involvement Working Group of the NIHR Oxford BRC and BRU), it is important to note: while timely publication of trials on registries and in professional publications is paramount, we are also working to identify how best to create lay-friendly information for those seeking information about trials and their outcomes, and how best to ensure it is made available to them.^18^

### Implications for practice

**Figure 5:**
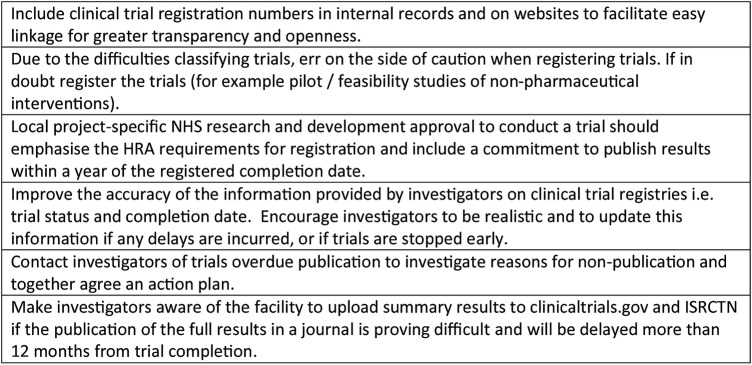
Organisational recommendations following the audit findings. HRA, Health Research Authority; NHS, National Health Service.

Based on the audit findings, the audit team and BRC/BRU Directors produced a set of recommendations to improve the transparency regarding which clinical trials receive BRC/BRU support and to encourage their timely publication (figure 5). The BRC/BRU Directors have committed to improving performance in these areas, and we are confident that with some simple changes and renewed prioritisation, this can be achieved.

**Figure 6:**
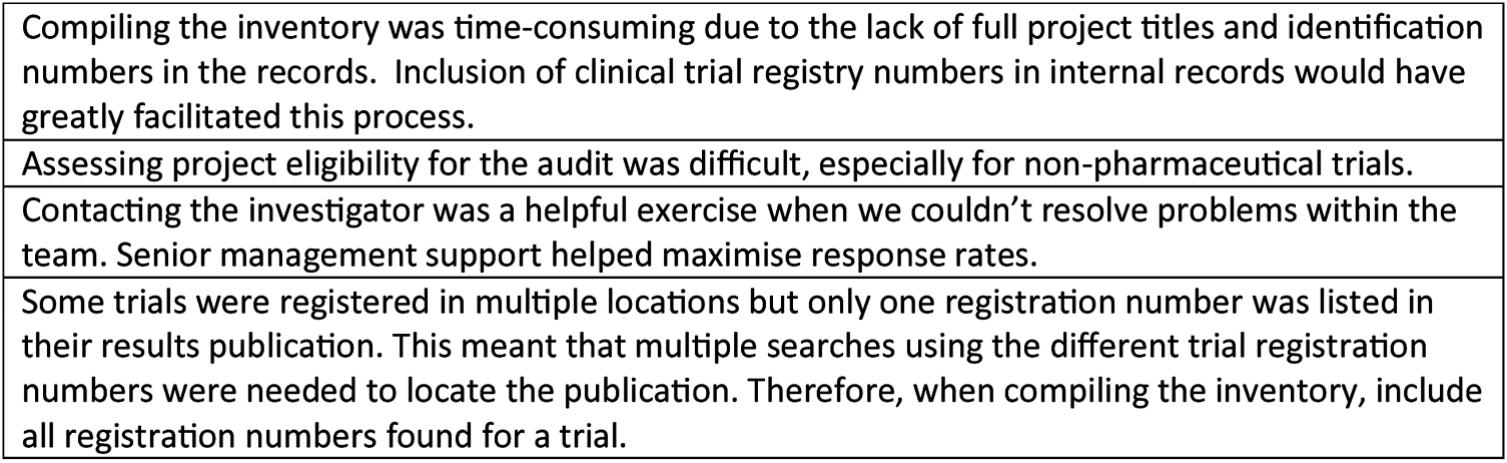
Learning points arising from conducting the audit.

More widely, routine ongoing audit has been proposed as a method by which institutions, sponsors and research funders can monitor their performance on registering and publishing trials, and use this to improve standards.^19^ ^20^ We have demonstrated that this is feasible and encourage other organisations to do the same. Our full methods (including template audit materials and analytic code) are available as an online supplementary appendix. Figure 6 describes what we have learnt having conducted the audit.

10.1136/bmjopen-2015-009285.supp1 Supplementary appendix

We are also aware of several local audits that have been conducted by various organisations since the current wave of renewed interest in trials transparency began. These include audits by sections of the Health Research Authority; the NIHR; and the Medical Research Council (to produce an estimate of publication bias for a 2012 UK parliamentary inquiry into trials transparency).^21^ To date, these audits all remain unpublished: we would welcome others sharing their methods, findings and insights. The major resource required for conducting our audit was the manual collection of data, cross-checking different sources and seeking clarification from individual researchers. If registry entries were consistently completed, and the information reliably maintained, it would be possible to automate almost the entire process of the audit. It would also then be trivial to derive—and display—an updated monthly performance metric, generated automatically by analytic code running on registry data. If all registry data at all institutions were maintained, this would in turn provide a ‘transparency dashboard’ on timely publication—a universally recognised metric of transparency—for easy comparison of different institutions, funders, drugs, principal investigators and fields of medicine.
