# Supplementary material for: Getting our house in order: an audit of the registration and publication of clinical trials supported by the National Institute for Health Research Oxford Biomedical Research Centre and the Musculoskeletal Biomedical Research Unit
Source: BMJ Open. 2016 Mar 2;6(3):e009285. doi: 10.1136/bmjopen-2015-009285 (PMC4785314; doi:10.1136/bmjopen-2015-009285)
Supplement: Supplementary appendix [file bmjopen-2015-009285supp.pdf]

How to conduct an audit of your own institution's registration and publication of clinical trials.

The template excel sheet is available here:
http://figshare.com/articles/Oxford_Trial_Audit_data_collection_template/1558323

Step 1: Creating the inventory of clinical trials
Use your internal records to populate the worksheet "eligibility tracking". Remove any duplicate entries. For each project try to determine if it is a phase 2-4 clinical trial and therefore eligible for inclusion in your audit. We found it was necessary to supplement information from our internal records with other sources to make a decision.

Clinicaltrials.gov, *https://clinicaltrials.gov/*
ISRCTN, *http://isrctn.org*
EuCTR, *https://www.clinicaltrialsregister.eu/ctr-search/search*
International Clinical Trials Registry Platform, *http://apps.who.int/trialsearch/*
UK Clinical Trials Gateway, *http://www.ukctg.nihr.ac.uk/default.aspx*
UK Clinical Research Network Study Portfolio, *http://public.ukcrn.org.uk/search/*
Health Research Authority, *http://www.nres.nhs.uk/researchsummaries/*
Pubmed, *http://www.ncbi.nlm.nih.gov/pubmed*

For any projects that you are unsure about, ask a second opinion from another member of your audit team and document in the "eligibility tracking" worksheet. If it still not possible to resolve eligibility, contact the lead investigator for the project at your institution using "template email A" (at the end of this document).

If your internal records are of good quality, it should be relatively straightforward to resolve eligibility and it may not be necessary to use other data sources/ contact the investigators.

Step 2: Populating the inventory and assessing registration
For each eligible project, complete a row in the "inventory" worksheet**.** Ignore the column headings greyed out in the spreadsheet (these are for later, optional analyses steps).

Data source	Describes which internal record was used to identify the project
Study Title, Acronym From Internal Records	This may not be the full registered title.

If your internal records contain trial registration numbers then it is easy to locate their register entry – simply type it into an internet search engine. If not, search the four major clinical trial registries (Clinical trials.gov, ISRCTN, EUCTR, ICTRP). If the full project title is not available from the internal records use combinations of other identifying features (e.g. acronym, drug name and target condition). Stick to the sequence of registers listed above for searching. Whenever possible use data from Clinicaltrials.gov, or if not available ISRCTN, to populate the worksheet as this contains the most information required for the audit. Once you have found and populated the worksheet with information from a register, you don't need to repeat searching and extracting data for the same trial from the other registers.

For each trial enter in the "inventory" worksheet:

Official title from Register	
Ref Number	This will be a unique identifier and can be used to check the inventory does not contain duplicate entries.
Status	For example “completed” or “discontinued”
Phase	
Sample Size	
Sponsor	Enter the institution here. For multicentre trials this will may not be your host institution.
Primary Completion Date	This is available for clinicaltrials.gov only.
Overall Completion Date	For EuCTR this is only available retrospectively
Results uploaded (Y/N)	
Date results uploaded	
Publication details present	Not available for EuCTR.

If it is not possible to locate a registration entry for a clinical trial, contact the lead investigator for the trial based at your institution using “Template Email A”. Manage the process using the “registration” worksheet. If registration details are provided by the investigator add these to the inventory.

Step 3: Verification of the inventory:

Check the completeness of your inventory using an alternative data source. We used a list of research publications that acknowledged BRC/U support and annual reports to see if we identified additional projects. We repeated steps 1 and 2 using these data sources and a new spreadsheet. We produced a list of trials that we cross-checked against our inventory using the ref number field to identify additional trials. We then added these trials to our original spreadsheet. The data source field was used to record that they were identified via this verification step.

Step 4: Assessing publication

This step applies to trials with a completion date of at least one year prior to the date of your audit. For trials registered on clinicaltrials.gov, use the overall study completion date; if not entered use the primary outcome completion date instead.

Do not include publications describing protocol design or analysis plans in this step of the audit. In addition to publications listed on the registry, we also searched PubMed (http://www.ncbi.nlm.nih.gov/pubmed) for articles arising from the completed trials.

Complete the following fields in the inventory worksheet.

Pubmed or other link	
Date	Use the e-publication date where available.

If it is not possible to locate a publication, contact the investigator using “Template Email B”. Manage this process using the “publications searching” worksheet. Add any publication details supplied by the investigator to the inventory worksheet.

Data analysis

To determine the number of trials overdue publication, complete the following fields of the inventory worksheet:

Dates used in calculation	Overall completion date	Depending on which clinical trial register used, this will be the date listed in column J or S or AB or AJ
	Primary outcome completion date	Column I (clinicaltrials.gov only)
	Publication due? (Y/N)	Column AS (or AT if not available) > 1 year from date of audit.
Publication delay	Publag (overall completion date)	Column AQ-AS
	Publag (primary outcome completion date)	Column AQ-AT
Overdue	Publication (Unpublished, overall trial completion date> 12 months from audit date) (Y/N)	Column AU = Y, and column AP = blank.
	Register results (Unpublished, overall trial completion date> 12 months from audit date) (Y/N)	Column AU = Y, and column K, T, AC, AK blank.
	Both (no results on register & not published)	Column AX and AY = "Y"

Classify the sponsoring organisation to complete the inventory worksheet:

Sponsor type coded	Code based on column H/Q/Z/AH<br>Either industry or non-industry
Own institution sponsor	Code yes/no

Collate the information collected on the trial characteristics from the different clinical trials register so that it is in one column and easier to analyse:

Phase	Column F or O or X or AF
Sample size	Column G or P or Y or AG
Status	Column E or R or AA or AI

Logistic regression

We used Stata to examine trial characteristics associated with slow/non-publication. You will need to save a copy of your finalised inventory as a CSV file to be able to import the data into this software. Delete the first two rows of column headings, leaving only the stata descriptors.

Here is the stata code to replicate our analysis.

STATA CODE

```
/*

STATA CODE FOR TRIAL PUBLICATION AUDIT
There are some places where the code will need to be amended for your audit data, especially
where "encode" has been used for speed.

*/
```

```
* ADDITIONAL PACKAGES REQUIRED
ssc install estout, replace
* (this package is very useful for exporting regression output)

* HOUSEKEEPING *
clear all
set more off, perm
cd YOURWORKINGDIRECTORY
insheet using YOURFILENAME.csv, clear names
save auditdata, replace
describe
browse

* make dates work in stata
gen pubdatecoded = date(pubdate, "DMY")
format pubdatecoded %td
gen completiondatecoded = date(dateusedincalculations, "DMY")
format completiondatecoded %td

* make variables

* make published yes no
gen pubyesno = pubdatecoded
recode pubyesno .=0 1/99999=1
tab pubyesno

* check the above
encode duepublicationas112015, gen (duepubcoded)
tab pubyesno duepubcoded

/*
* THIS code will need to be rewritten after examining your own data
* make phase variable
encode phase, gen (phasecoded)
tab phasecoded
* CLEAN YOUR PHASE CODING
recode phasecoded * YOUR CODE HERE
*/

* drop irrelevant records
* recommend keeping track of counts when doing drops
count
drop if dateusedincalculations=="Not registered"
```

```
count

* drop if completion date is such that this trial is outside of your inclusion criteria
* THIS DATE WILL NEED TO BE AMENDED BASED ON YOUR STUDY INCLUSION DATES
drop if completiondatecoded > 20088
* 20089 comes from * di date("20150101","YMD")
count

*pubdelay varibale
gen pubdelay = completiondatecoded-pubdatecoded

* make status variable
capture drop statuscoded
encode status, gen (statuscoded)
tab statuscoded
tab statuscoded, nolabel
/* THIS CODE WILL NEED TO BE AMENDED FOR YOUR STUDY TO ENSURE RELEVANT DROPS
drop if statuscoded ==
*/

* make industry yes / no variable
capture drop sponsorcoded
encode sponsortypecoded, gen (sponsorcoded)
tab sponsorcoded
tab sponsorcoded, nolabel
capture drop industryyesno
gen industryyesno = sponsorcoded
* THIS CODE WILL NEED TO BE REWRITTEN AFTER EXAMINING YOUR OWN DATA
recode industryyesno * YOUR CODE HERE
tab industryyesno sponsorcoded

* make size (n) quartiles
gen n = real(samplesize)
egen samplesizequartile=cut(n), group(4) label
tab samplesizequartile

* regression analysis and tables
* CODE ABOVE TO INSTALL ESTOUT
estimates clear
eststo clear
logistic pubyesno ib(freq).industryyesno, allbase
eststo
logistic pubyesno ib(freq).phasecoded, allbase
eststo
logistic pubyesno ib(freq).samplesizequartile, allbase
```

```
eststo
logistic pubyesno ib(freq).phasecoded ib(freq).industryyesno ib(freq).samplesizequartile, allbase
eststo
estout using audittables.xls, cells ("b(fmt(2)) ci p(fmt(3))") eform replace

* tables comparing published vs unpublished trials
tab pubyesno industryyesno, col chi
tab pubyesno phasecoded, col chi
tab pubyesno samplesizequartile, col chi
```

Template Email A (email to resolve eligibility)

Dear Professor/Dr [Name],

As part of the [organisation's name] commitment to transparency, we are conducting an audit of the clinical trials that have received [organisation's name] support. This aim of the audit is to assess rates of registration and publication, and has the support of the [organisation's name] senior management.

From the records kept by the [organisation's name], we have identified your following project:
"Study title"

1. Please could you provide the full study title?
2. Please could you confirm if this is a phase 2-4 clinical trial?
2a. If so, please could you provide the clinical trial registration numbers (e.g. clinical trials.gov, ISCTRN, EudraCT)
3. Please could you provide details of any publications resulting from this project?

I would be very grateful if you could complete the table and return it to me by [insert date].

If you have any questions or would like further information about the audit, please contact [provide phone, email address]

Thank you in advance for your help.

Yours sincerely,

XXX, on behalf of the audit team:

Template Email B (email to resolve publication status)

Dear Professor/Dr [Name],

As part of the [organisation's name] commitment to transparency, we are conducting an audit of the clinical trials that have received [organisation's name] support. This aim of the audit is to assess rates of registration and publication, and has the support of the [organisation's name] senior management.

From the records kept by the [organisation's name], we have identified your following project:

"Trial title"

According to its entry on the [Clinical trial.gov/ISRCTN] registry [Clinical registration number], the trial had a completion date of [date].

1. Have the results of this clinical trial been published?
2. If yes, please provide the publication details.
3. If no, please provide details of what has delayed/ prevented publication

I would be very grateful if you could complete the table and return it to me by [insert date].

If you have any questions or would like further information about the audit, please contact [provide phone, email address]

Thank you in advance for your help.

Yours sincerely,

XXX, on behalf of the audit team:
